# Vacuum-assisted excision of breast lesions in surgical de-escalation: where are we?

**DOI:** 10.1590/0100-3984.2022.0078-en

**Published:** 2023

**Authors:** Beatriz Medicis Maranhão Miranda, Almir Galvão Vieira Bitencourt

**Affiliations:** 1 Breast Center, Instituto de Medicina Integral Prof. Fernando Figueira - IMIP, Recife, PE. Brazil; 2 Lucilo Maranhão Diagnósticos, Recife, PE, Brazil; 3 Department of Imaging, A.C.Camargo Cancer Center, São Paulo, SP, Brazil; 4 DASA, São Paulo, SP, Brazil

**Keywords:** Breast neoplasms, Image-guided biopsy, Biopsy, needle, Minimally invasive surgical procedures, Neoplasias da mama, Biópsia guiada por imagem, Biópsia por agulha, Procedimentos cirúrgicos minimamente invasivos

## Abstract

Vacuum-assisted excision of breast lesions has come to be widely used in clinical practice. Increased acceptance and availability of the procedure, together with the use of larger needles, has allowed the removal of a greater amount of sample, substantially reducing the surgical upgrade rate and thus increasing the reliability of the results of the procedure. These characteristics result in the potential for surgical de-escalation in selected cases and gain strength in a scenario in which the aim is to reduce costs, as well as the rates of underestimation and overtreatment, without compromising the quality of patient care. The objective of this article is to review the technical parameters and current clinical indications for performing vacuum-assisted excision of breast lesions.

## Abstract (pt)

A excisão assistida a vácuo de lesões mamárias tem sido cada vez mais utilizada na prática clínica. A sua maior aceitação e disponibilidade, em associação ao uso de agulhas mais calibrosas, permitiu a retirada de quantidade maior de amostra, reduzindo substancialmente a taxa de subestimação diagnóstica e aumentando, assim, a confiabilidade final dos resultados do procedimento. Essas características resultam em potencial descalonamento cirúrgico, em casos selecionados, e ganham força em um cenário em que se visa a redução de custos, taxa de subestimação e tratamento excessivo, porém, sem comprometer a qualidade no cuidado com o paciente. O objetivo deste trabalho é revisar os parâmetros técnicos e as indicações clínicas atuais para realização de excisão assistida a vácuo em lesões mamárias.

## INTRODUCTION

In 1995, vacuum-assisted biopsy (VAB) was introduced as a percutaneous diagnostic method for breast lesions and was initially performed with a 14G needle. As of 2010, studies began to report the possibility of excising lesions using this method, either as a secondary benefit or as an initial indication, referring to it as vacuum-assisted excision (VAE). Since then, VAE has been ever more widely used in clinical practice. The greater acceptance and broader availability, together with the use of larger caliber needles, has allowed the removal of a larger amount of sample, substantially reducing the rate of diagnostic underestimation and thus increasing the reliability of the results of the procedure^(1)^. This results in potential surgical de-escalation, reducing the extent of the surgical intervention in selected cases, and gains strength in a scenario in which the aim is to reduce costs, as well as the rates of underestimation and overtreatment, without compromising the quality of patient care.

It is crucial for interventional radiologists to understand the current scenario and the potential applications of VAE, because it can change the clinical management of some breast lesions by updating practices over the years. The objective of this article is to review the technical parameters and current clinical indications for VAE of breast lesions.

## TECHNICAL CONSIDERATIONS

The use of VAE begins with the formation of an integrated multidisciplinary team, in which the interventional radiologist is responsible for ensuring that the procedure is performed safely and judiciously, under the close supervision of the breast surgeon, with well-defined eligibility criteria and objectives, and that the excised sample is evaluated in detail by the pathologist. When performing a vacuum-assisted procedure, it is important to be explicit about the purpose of the procedure in question. The United Kingdom’s National Health Service recommends using specific codes and consent forms for the various types of vacuum-assisted procedures^(2)^, stating that the focus of VAB, the use of which is quite widespread, should be solely on diagnosis, rather than on removing the lesion completely, whereas VAE, the aim of which is to be a substitute for a diagnostic surgical biopsy and which might not be the first procedure the patient has undergone, should be focused on removing the lesion in its entirety.

**Figure 1:**
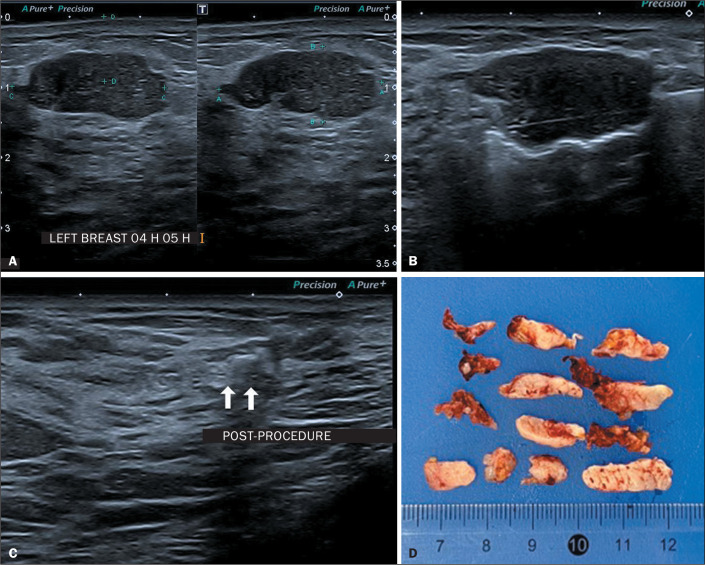
Patient with a core biopsy diagnosis of a fibroadenoma, initially measuring 1.2 cm, thereafter presenting growth and becoming palpable finding, growing to 2.3 cm by six months after diagnosis, when it was submitted to VAE. A: Pre-excision ultrasound showing the target lesion. B: Ultrasound during the procedure, showing the positioning of the needle below the lesion and activation of the vacuum. C: Post-excision ultrasound showing the clip marking the biopsy site. D: Macroscopic result of the fragments obtained from excision with a 7G needle.

The size of the lesion to be submitted to the procedure matters, because lesions larger than 1.5 cm are not easily excised. However, Park et al.^(3)^ argued that there should be no size limit for excision, which also depends on the location of the lesion, its relationship with adjacent structures, patient comfort, and the risk of complications related to VAE for lesions larger than 3.0 cm. Benign nodules that show growth can be submitted to VAE (Figure 1).

**Figure 2:**
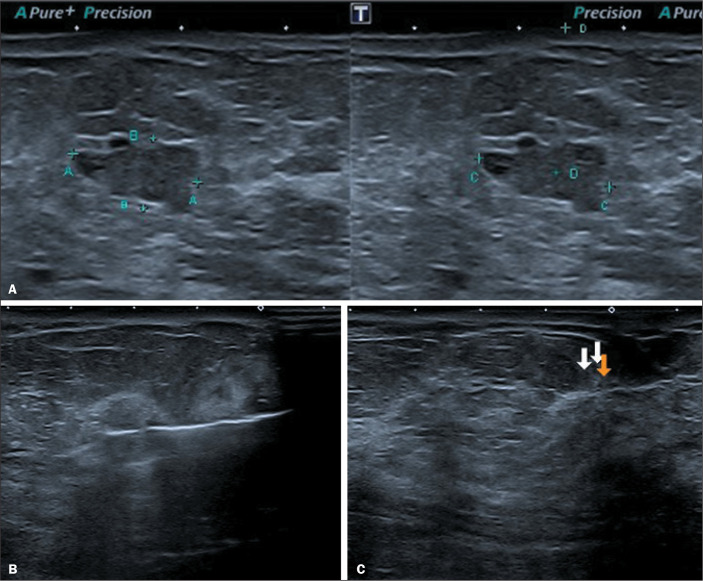
Patient with a core biopsy diagnosis of a papillary lesion, subsequently submitted to VAE. A: Pre-excision ultrasound showing the target lesion. B: Ultrasound during the procedure, showing the positioning of the needle below the lesion and activation of the vacuum. C: Post-excision ultrasound showing the clip marking the biopsy site (arrows). The histological result was consistent with intraductal papilloma with a focus of atypical epithelial proliferation, measuring 3.5 mm, demonstrating the presence, by quantitative criteria, of intraductal papilloma with low-grade ductal carcinoma in situ.

In VAB, the number of specimens to be collected will be directly proportional to the size of the lesion. Dekker et al.^(1)^ suggested the following: obtain six specimens with a 9G needle, from the lesion and from the periphery, in order to have 95% accuracy in the final diagnosis. In VAE, oblique aspiration should be avoided; rather, the lesion should be removed orthogonally, in order to obtain the largest possible specimen, which is an important factor in determining the number of atypical features in the lesion, given that the histological criterion to differentiate atypical ductal hyperplasia from ductal carcinoma *in situ* is quantitative and has the aim of reducing the rate of diagnostic underestimation. One scenario in which VAE is indicated is when a core biopsy has resulted in a histological diagnosis of a papillary lesion (Figure 2).

**Figure 3:**
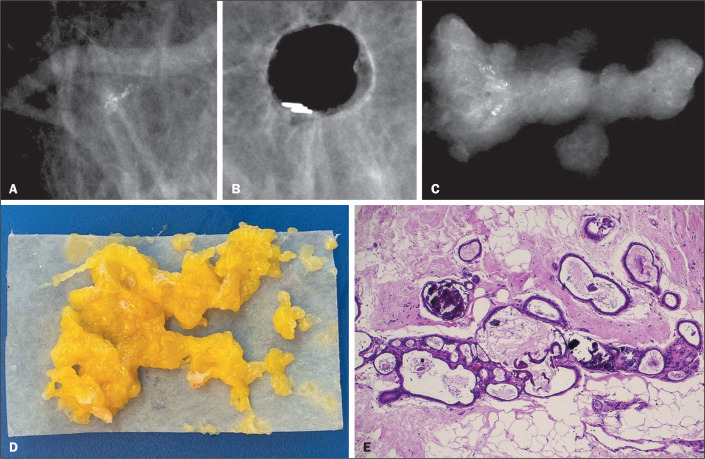
Clustered microcalcifications (A) that were subsequent excised completely, a metal clip being inserted to mark the biopsy site (B). A radiograph of the specimens (C) and a photograph of their macroscopic aspect (D), the results being consistent with ductal calcifications without atypia, which was confirmed in the histological examination (E).

One of the most important points in VAE is to have immediate control after the procedure, in order to confirm the completeness of the excision and the absence of residual lesion, which makes it mandatory to monitor the patient and to obtain radiological confirmation at the end of the procedure. If the procedure is guided by ultrasound, the monitoring is performed in real time. In stereotactically guided VAE, it is necessary to look for residual calcifications at the biopsy site and to verify the inclusion of the lesion in the samples obtained, through radiography of the specimens and histopathological examination, in order to determine the radiological-pathological correlation (Figure 3).

## INDICATIONS

To date, vacuum excision has had numerous applications: for excision of previously biopsied lesions with a histological diagnosis of high risk or uncertain malignant potential (B3 lesions); for exeresis of benign lesions when the procedure has been requested by a clinician; and for a repeat biopsy in cases in which there is discordance between the radiological and pathological findings. The potential applications of VAE have been described in recent studies. For example, one study described the use of VAE for the excision of gynecomastia^(4)^. There are also ongoing studies on the potential applicability of vacuum excision of the tumor bed after neoadjuvant chemotherapy in selected cases (those showing a response on imaging), as well as on that of exeresis as a minimally invasive alternative to conventional surgery for small, incipient, biologically favorable tumors detected by screening^(5,6)^.

In a study comparing the cost of VAE with that of open surgery, VAE was found to be the less costly of the two, for benign lesions and for high-risk lesions, even when the cost of the follow-up imaging evaluations was taken into account^(2)^. The authors concluded that greater utilization of VAE in selected cases could reduce health costs and avoid unnecessary surgical procedures.

### Lesions of uncertain malignant potential

High-risk lesions, which are detected in 4-9% of biopsies, constitute a heterogeneous group of lesions with uncertain malignant potential, given the risk of underestimation in the surgical upgrade, and can be precursor lesions with the potential to progress to invasive carcinoma^(4)^. Because it allows a definitive diagnosis of cancer to be made in patients scheduled to undergo surgery for conditions other than cancer, VAE can play a diagnostic role by identifying malignancy in the preoperative period, thus ensuring the correct surgical management of such cases in a single procedure. Data in the literature underscore the need for the alternative management of B3 lesions and suggest that VAE is appropriate in selected cases^(2,4,7-11)^. For VAE of high-risk breast lesions, the United Kingdom’s National Health Service recommends obtaining 4 g of material, the number of fragments depending on the caliber of the needle used^(2)^, as detailed in Table 1.

**Table 1:** Mean number of fragments needed to achieve approximately 4 g of tissue in the VAE procedure for devices currently available on the market.^*^

Device	Needle gauge	Fragment weight Mean ± SD	Mean number of fragments needed
EnCore Enspire^†^	10G	0.221 ± 0.039	18
	7G	0.363 ± 0.053	11
ATEC Sapphire^‡^	9G	0.121 ± 0.014	33
Mammotome Revolve^§^	8G	0.334 ± 0.046	12

* Adapted from Pinder et al.^(2)^.

† Bard Biopsy Systems, Tempe, AZ, USA.

‡ Hologic Inc., Marlborough, MA, USA.

§ Devicor Medical Products Inc., Cincinnati, OH, USA.

In 2019, Shaaban et al.^(8)^ suggested that radial scarring without atypia and papillary lesions without atypia should be treated by vacuum excision, and that, in cases in which VAE was performed after VAB, it was mandatory to identify, histologically, the center of the previous biopsy and the reaction to the clip, in order to have a reliable correlation with the upgrade of the lesion removed. The authors stated that, based on the specimen fragments sampled, the pathologist could not comment on whether there was complete excision of the lesion and its margins, the confirmation of which would depend on the radiological impression. However, in clinical practice, some groups have tried to overcome this limitation by sending the material to pathology in separate vials, one with the fragments that contain the lesion and one with the fragments that include the margins removed in a 360-degree resection around the lesion. Studies have shown that VAE is safe in cases of papillary lesions without atypia, presenting a rate of upgrade to carcinoma of 0%, compared with approximately 10% for core biopsy^(12,13)^.

Since the first international consensus conference on lesions of uncertain malignant potential (B3 lesions), in 2016, to the present day, the approaches to such lesions have been constantly readjusted^(9)^. In 2021, Catanzariti et al.^(10)^ suggested that VAE could be used in cases of papillary lesions without atypia; radial scarring with or without atypia; flat epithelial atypia; and lobular neoplasia (which includes atypical lobular hyperplasia and lobular carcinoma *in situ*). However, for cases of atypical intraductal epithelial proliferation, the authors suggested that VAE be used only in cases of small lesions with atypical ductal hyperplasia that is unifocal.

When there is lesion present in the margins evaluated, it is important to determine the radiological-pathological correlation. In the case of flat epithelial atypia, given its mild relative risk-1 to 1.5 times greater than that of breast cancer, similar to that attributed to typical usual ductal hyperplasia-there is no recommendation in the literature for tumor-free margins if there are no other lesions with significant pathology, such as atypical ductal hyperplasia and atypical lobular hyperplasia/lobular carcinoma *in situ*, and residual microcalcifications should always be excised after the procedure^(14)^.

The United Kingdom’s National Health Service guidelines for the management of B3 lesions state that, in order to adopt the appropriate management, those submitted to core biopsy, fine needle aspiration, or first-line VAB can be referred for VAE as a second-line procedure. In such cases, the following protocol is followed, depending on the histological diagnosis^(2,4)^: if it is a benign lesion without atypia, the patient is referred for regular follow-up; if it is a lesion with focal atypia, the patient is followed, under active surveillance of the biopsied region, for a period of five years, including evaluation by imaging methods, such as magnetic resonance imaging, in selected cases; and if it is a malignant lesion, the patient is referred for definitive, therapeutic surgery, diagnostic surgery therefore being precluded. To select the patients to be followed, each case must be assessed for commitment to follow-up^(11)^, taking into account factors such as the age and risk level of the patient; the size of the lesion and its relation to the volume removed; whether there is residual lesion; and whether the lesion in question was a primary or incidental histological finding.

It should be borne in mind that surgery is still the rule in cases of atypical ductal hyperplasia; pleomorphic lobular carcinoma *in situ*; extensive lobular carcinoma *in situ* with necrosis or accompanied by other high-risk lesions; the combination of flat epithelial atypia and atypical ductal hyperplasia; and palpable papillary lesion with atypia, papillary flow, and calcifications. On the basis of the current knowledge, VAE can be indicated in cases of papillary lesion without atypia, radial scarring, flat epithelial atypia, unifocal atypical ductal hyperplasia, and classical atypical lobular hyperplasia/lobular carcinoma *in situ*, especially when identified as incidental findings^(10)^.

### Benign lesions

For the vacuum excision of benign lesions, VAE has the advantage of greater affordability in comparison with the surgical procedure, which would involve the following^(15)^: hospital admission; induction of anesthesia, medical and hospital materials; preoperative localization; and considerable time spent in preoperative visits. In addition, the postprocedure aesthetics and the prolonged recovery time after open surgery result in lower patient satisfaction^(15)^. The main clinical indications for VAE are low adherence to follow-up, patient anxiety, increase in lesion volume during follow-up, symptoms, and planning to start hormone replacement therapy for assisted reproduction^(16,17)^.

For nodules that show rapid growth, such as phyllodes tumors, studies show that the post-VAE recurrence rate is 5-17% in benign cases when 3.3 cm is used as the lesion size cutoff point^(18)^. In addition, the rate of recurrence is lower for lesions smaller than 1.5 cm, making VAE an alternative to surgery for the complete excision of benign tumors. In cases in which the results of previous biopsies were discordant, it can be advantageous to use VAE because it allows the entire lesion to be aspirated, thus avoiding a lesion sampling error, as often occurs in complex solid-cystic lesions. In cases of mild gynecomastia, not requiring surgical reduction of the skin or of the nipple-areolar complex, VAE is considered a viable alternative to the classic surgical procedure for gynecomastia, resulting in smaller scars and shorter hospital stays, with similar aesthetic results^(19,20)^.

### Malignant lesions

Among the ongoing studies of the potential applications of VAE^(5,6)^, one notable effort is the RESPONDER clinical trial^(5)^, which was designed to evaluate the accuracy of VAB for the diagnosis of a pathological complete response-defined as the absence of residual lesion in the tumor bed-after preoperative neoadjuvant treatment, given that, depending on the molecular subtype, a pathological complete response can be achieved in up to 60% of patients with breast cancer. However, the accuracy of imaging evaluation after neoadjuvant treatment can be limited and surgery is therefore considered mandatory at the end of treatment, in order to remove residual disease or histologically diagnose the treatment response. Since the 2018 announcement of that trial, no results have been published, because of problems with patient recruitment and the difficulties provoked by the pandemic. The authors pointed out that the histopathological evaluation of a non-tumor sample is a critical issue, creating uncertainty as to whether the region of the tumor with a pathological complete response has been sampled or if unrepresentative tissue was removed, which could introduce a sampling error^(5)^. Similarly, the SMALL clinical trial was initiated in the context of a discussion about incipient, biologically favorable cancers diagnosed by screening and whether they could be treated by VAE, thus precluding surgery as well as minimizing overtreatment, morbidity and costs^(6)^. The authors applied the following eligibility criteria: being over 47 years of age; having had no previous breast tumor; and currently having a tumor that is smaller than 1.5 cm, contains no microcalcifications, is unifocal, is classified as grade 1, is strongly positive for estrogen/progesterone receptors, is negative for human epidermal growth factor receptor 2, and does not involve axillary lymph nodes.

**Figure 4:**
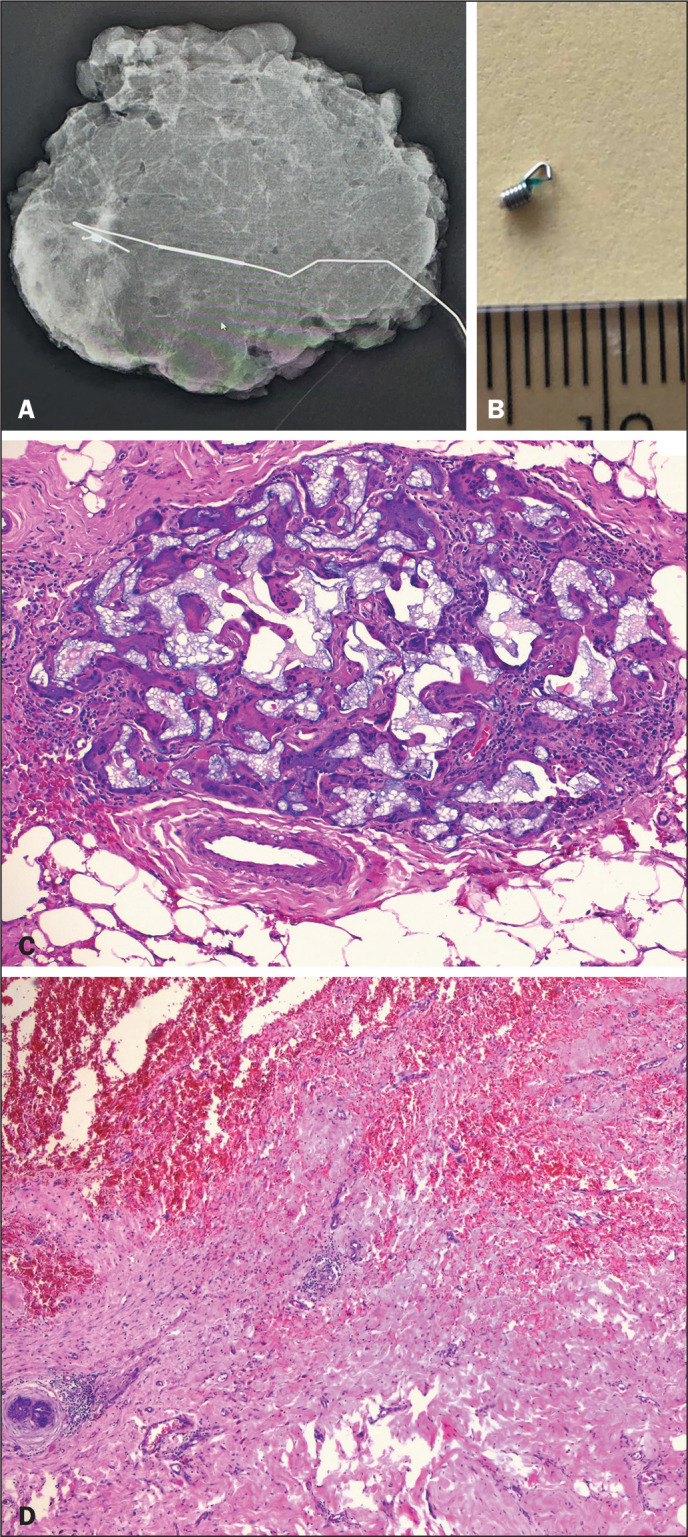
A: Radiograph of a surgical specimen containing a metal clip inserted after VAE, indicated with a metal wire. B: Metal clip. C,D: Histological sections of the specimen, showing the VAE site, fibrotic scarring, foci of recent hemorrhage, foreign body giant cell inflammatory reaction, and no residual neoplasia, the tumor diameter measured on the pre-VAE ultrasound being used in order to determine the size of tumor for staging purposes.

After surgical excision in patients submitted to VAE with a result of malignancy, the major axis of the infiltrative neoplasm-in the biopsy specimen and in the surgical specimen-should be measured on its longest axis and those data should be correlated with the prebiopsy imaging, in order to determine the size of the tumor for staging. If the tumor is small and there is no residual lesion in the resected specimen, the tumor-node-metastasis staging should consider the dimensions of the tumor as measured on the prebiopsy imaging, because of the fragmentation of the lesion, and it is important to send a detailed report with the correct documentation of the lesion and measurements before the procedure is performed (Figure 4).

## COMPLICATIONS

Post-VAE complications, which have been observed in various interventional breast procedures, include ecchymosis, infection, pseudoaneurysm, and pneumothorax, as well as others related to the vacuum procedure, such as hematoma, skin laceration, clip migration, scarring, postbiopsy distortion, and fat necrosis, with or without calcification. It is extremely important to know not only how to perform the procedure but also how to manage complications promptly, accurately, and resolutely, which is the responsibility of those who perform the procedure. Hematomas constitute the most common complication and can predispose to clip migration, together with rapid breast decompression. Skin laceration in the region can be avoided by using the Berná-Serna maneuver, which consists of fixing a cannula between the skin and the lesion, in order to prevent the skin from being suctioned when the vacuum system is activated^(21-24)^.

## CONCLUSIONS

The applicability of VAE relies on a multidisciplinary approach, with close communication between members of the multidisciplinary team regarding when to indicate the procedure, which complications are acceptable, what are the best practices after the procedure, and which follow-up regimen should be employed. In recent years, the technique has evolved considerably. Notably, the pathological results have improved, approaching those of sectorectomy/nodulectomy analyses, with the use of the information and measurements that are necessary for ensuring the reliability of the method. Such advances have led to greater popularization of VAE, resulting in surgical de-escalation in properly selected cases. However, interdisciplinary integration and synergy are vital for the success of the method.
